# Giant Clear Cell Renal Cell Carcinoma With Continuous Tumor Thrombus Extension Into the Left Renal, Gonadal, Ascending Lumbar, and Hemiazygos Veins: A Case Report

**DOI:** 10.7759/cureus.114050

**Published:** 2026-08-06

**Authors:** Salma Erguiague, Mohamed Ali Addou, Siham Chahboune, Asmae Oulad Amar, Siham Alaoui Rachidi

**Affiliations:** 1 Department of Radiology, Mohammed VI University Hospital, Faculty of Medicine and Pharmacy of Tangier, Abdelmalek Essaâdi University, Tangier, MAR

**Keywords:** clear cell renal cell carcinoma, gonadal vein, hemiazygos vein, renal vein, tumor thrombus

## Abstract

Venous tumor thrombus is a well-recognized feature of clear cell renal cell carcinoma (ccRCC), most commonly involving the renal vein and, in advanced cases, the inferior vena cava. However, simultaneous extension into multiple collateral venous pathways, particularly the gonadal and hemiazygos veins, is exceedingly uncommon. We report the case of a patient with a giant left-sided ccRCC measuring 20 cm, presenting with extensive venous tumor thrombus involving the left renal vein, the left gonadal vein, the left ascending lumbar vein, and the hemiazygos vein without direct inferior vena cava invasion. Contrast-enhanced computed tomography demonstrated complete distortion of the renal architecture, marked local mass effect on adjacent abdominal structures, encasement of the left renal artery, and extensive perirenal fat infiltration. The tumor thrombus reached the ostium of the left renal vein at the inferior vena cava while remaining confined to the renal venous system and collateral veins. Additional findings included delayed bilateral renal excretion, mild-to-moderate ascites, and indeterminate small hepatic lesions without significant retroperitoneal lymphadenopathy. Histopathological examination confirmed ccRCC. This case illustrates an exceptionally rare pattern of multivenous tumor thrombus involving the renal, gonadal, ascending lumbar, and hemiazygos veins. Comprehensive preoperative CT evaluation is essential for accurately delineating the extent of venous involvement, guiding therapeutic planning, and raising awareness of atypical collateral venous extension in advanced RCC.

## Introduction

Renal cell carcinoma (RCC) is the most common primary malignant renal malignancy and is characterized by a marked tendency for venous invasion, with tumor thrombus occurring in approximately 4-10% of cases. Venous extension most frequently involves the renal vein and inferior vena cava (IVC), making accurate preoperative assessment essential for staging, surgical planning, and prognosis [1,2]. Contrast-enhanced multidetector computed tomography (MDCT), particularly with multiplanar and maximum-intensity projection (MIP) reconstructions, is the imaging modality of choice for evaluating venous tumor thrombus and guiding surgical management [3]. Treatment depends on the extent of venous involvement and requires a multidisciplinary approach [4]. While renal vein and IVC invasion are well recognized, extension through collateral venous pathways remains exceptionally rare. The hemiazygos vein, which ascends along the left side of the thoracic spine and communicates with the left ascending lumbar vein before crossing to join the azygos vein, constitutes an important collateral venous pathway. Tumor extension through this route is exceptionally uncommon and has rarely been described [5-7].

## Case presentation

A 48-year-old woman with end-stage renal disease on maintenance hemodialysis presented with a several-month history of progressive left flank pain and abdominal distension. She also reported episodes of gross hematuria, which she had initially ignored, as well as progressive weight loss and deterioration of her general condition. Laboratory investigations revealed anemia and findings consistent with end-stage renal disease.

**Figure 1:**
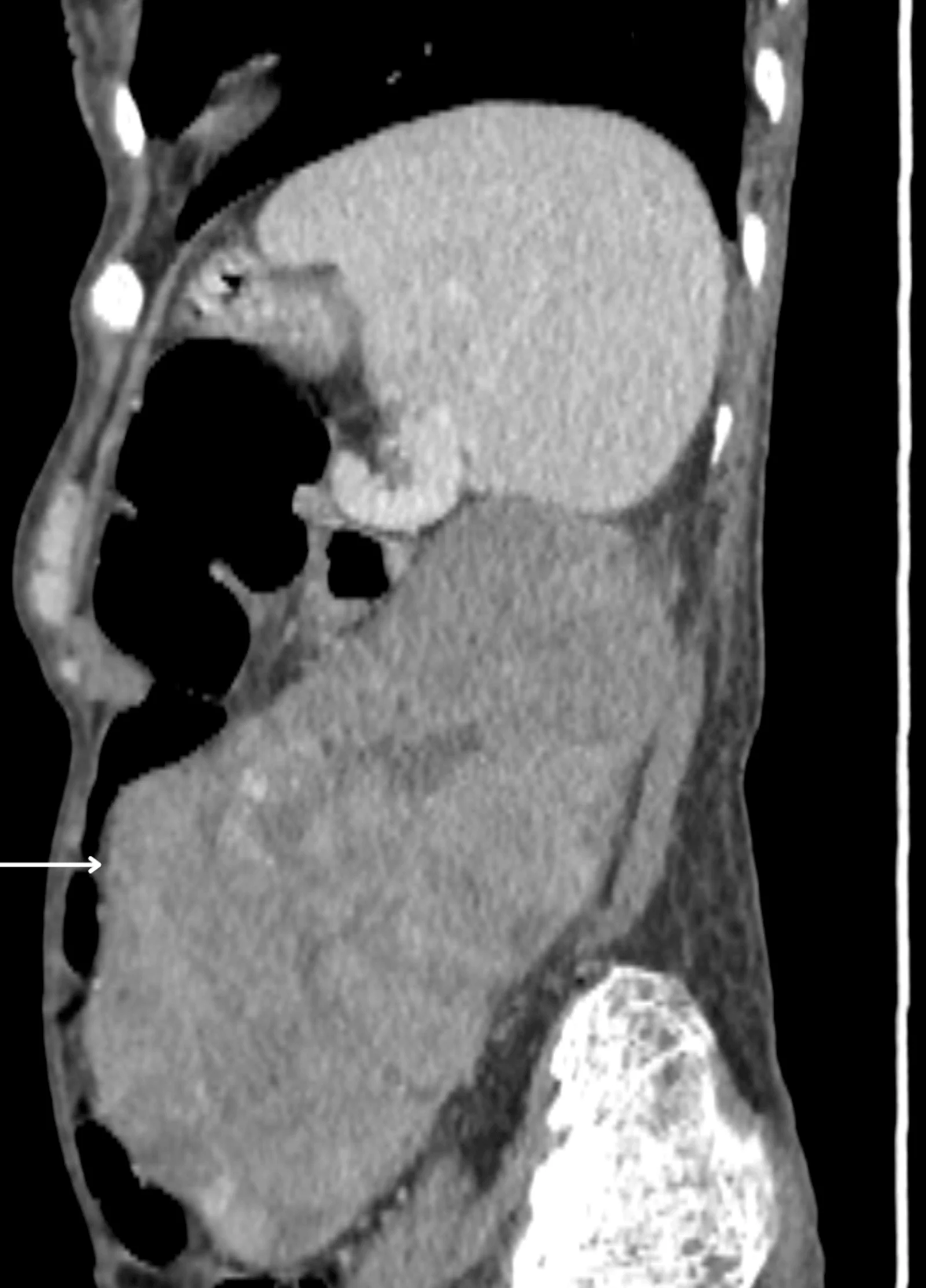
Sagittal contrast-enhanced CT image demonstrating a giant heterogeneous left renal mass with extensive central necrosis and marked mass effect on adjacent abdominal structures (arrow).

Contrast-enhanced abdominal CT revealed a giant heterogeneous left renal mass measuring approximately 20 × 10 × 10 cm, exhibiting heterogeneous contrast enhancement with extensive central necrosis (Figure 1). The lesion completely distorted the normal renal architecture, with obliteration of the renal sinus and pelvicalyceal system. Extensive perirenal fat infiltration was present, and the tumor demonstrated intimate contact with the spleen, descending and transverse colon, and left psoas muscle. Superiorly, it completely encased the left renal artery and its segmental branches, displaced the pancreas anteriorly, and deviated the abdominal aorta toward the right.

**Figure 2:**
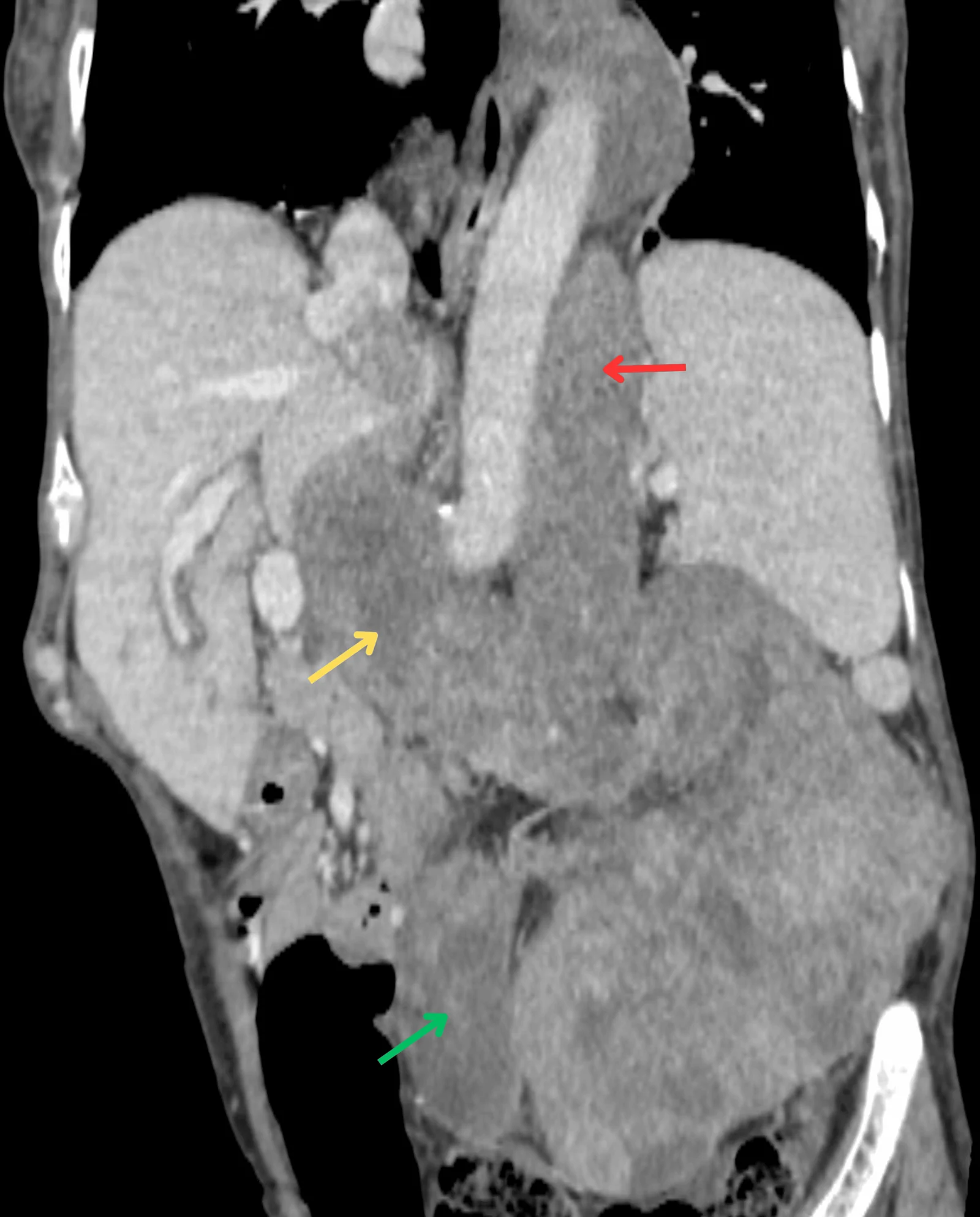
Coronal contrast-enhanced CT reconstruction demonstrating contiguous tumor thrombus extending from the left renal vein (yellow arrow) into the left gonadal vein (green arrow), with further cranial extension into the left ascending lumbar vein (red arrow).

**Figure 3:**
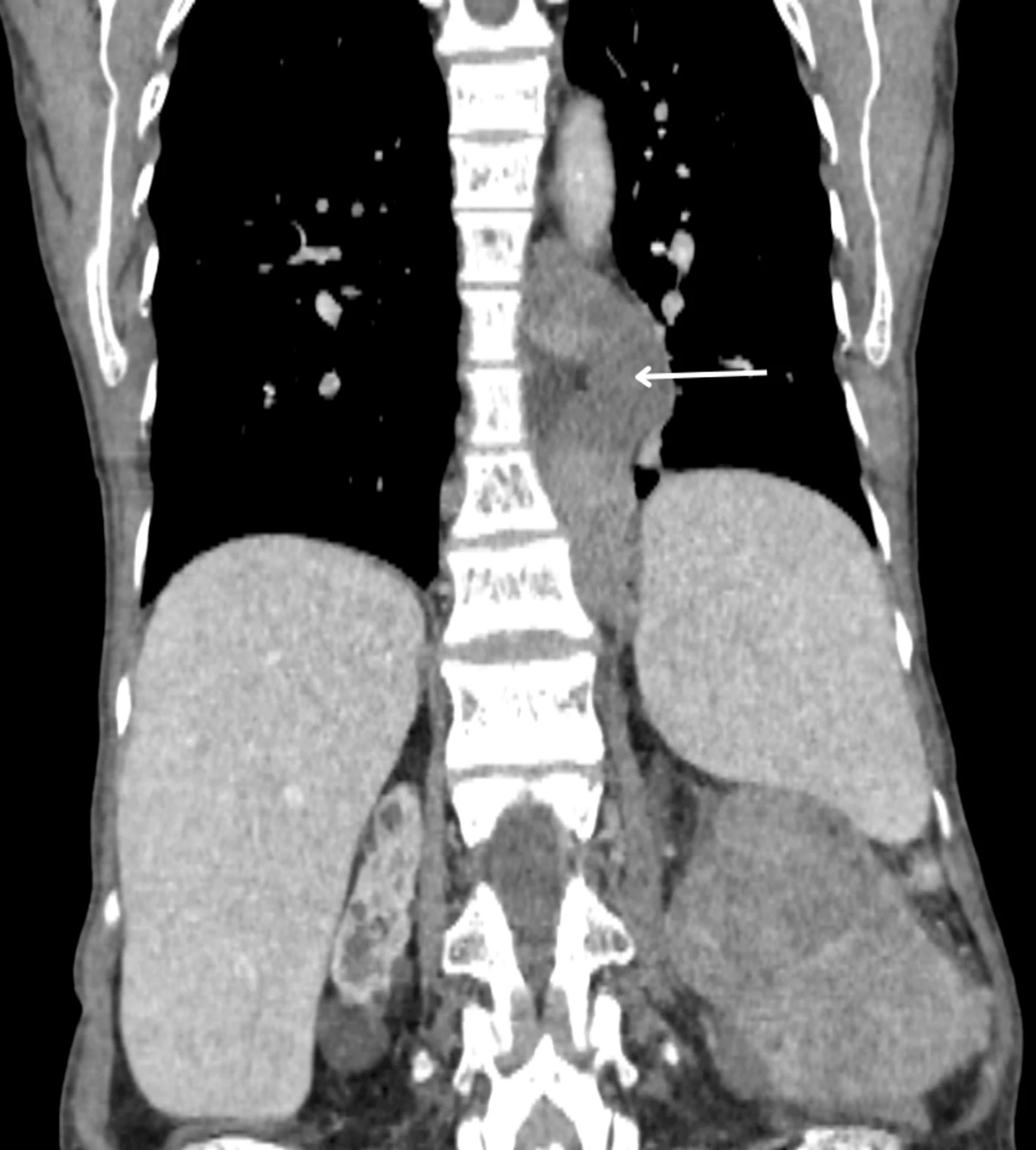
Coronal contrast-enhanced CT reconstruction obtained at a higher thoracic level demonstrating continued tumor thrombus within the left hemiazygos vein (white arrow).

A large enhancing tumor thrombus occupied the left renal vein, extending from the renal hilum to its ostium at the IVC, without evidence of caval invasion (Figure 2). The thrombus caused marked extrinsic compression of the portal vein, which remained patent. Contiguous tumor thrombus propagated into the left gonadal vein (Figure 2), with further cranial extension through the left ascending lumbar vein and into the hemiazygos vein (Figure 3), representing an exceptionally rare collateral venous route of tumor spread. The continuity of the enhancing intravascular soft-tissue component with the primary renal mass strongly supported the diagnosis of malignant venous tumor thrombus.

The contralateral kidney was markedly atrophic and contained several simple cortical cysts. No significant abdominal or retroperitoneal lymphadenopathy was identified. A small-to-moderate amount of intraperitoneal ascites was present.

**Figure 4:**
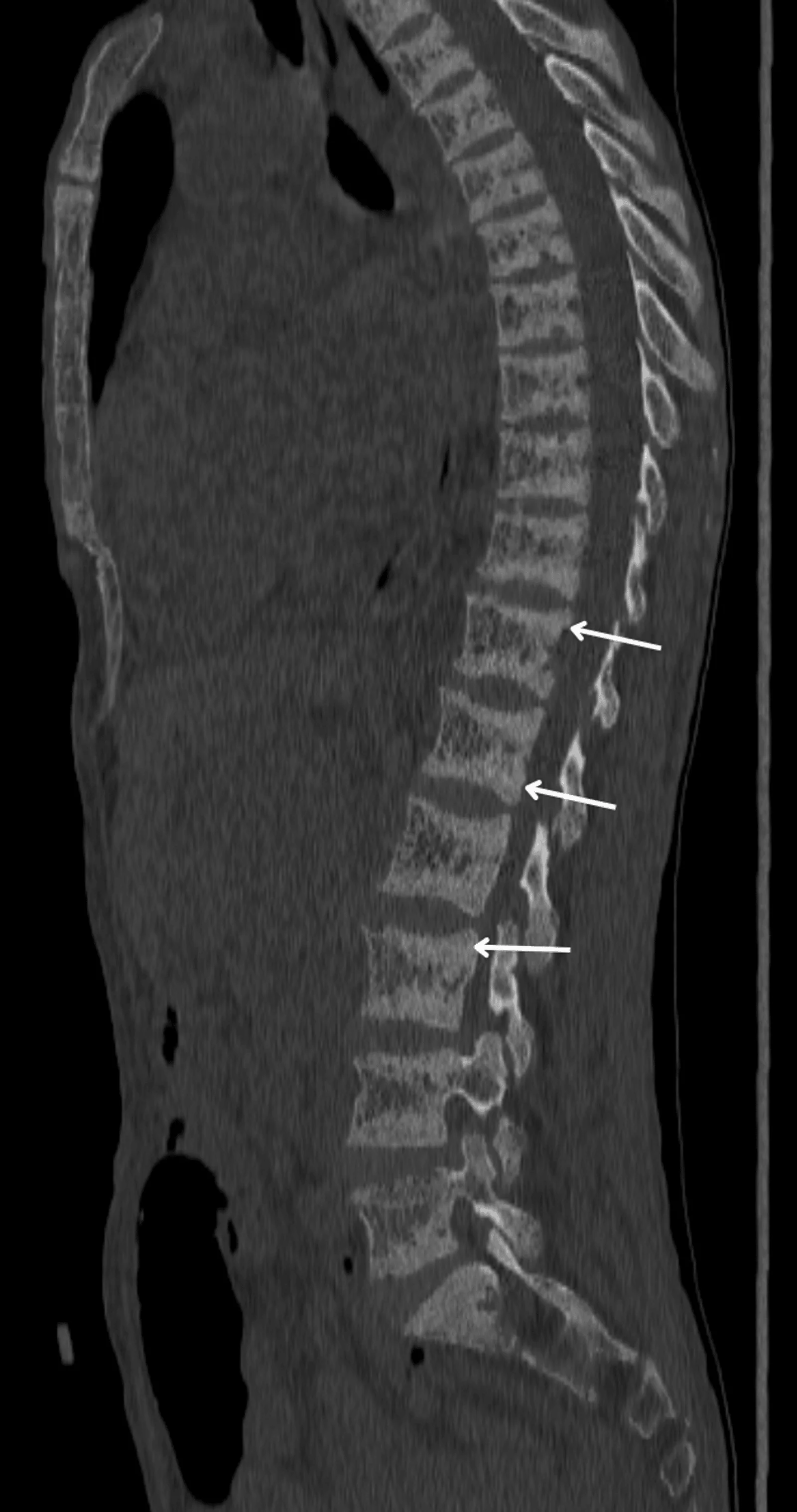
Sagittal bone-window CT image showing diffuse sclerosis of the superior and inferior vertebral endplates with relative central lucency, producing the characteristic rugger-jersey spine appearance (arrows), consistent with renal osteodystrophy secondary to chronic kidney disease.

Bone-window CT images demonstrated diffuse sclerosis of the superior and inferior vertebral endplates with relative central lucency, producing a characteristic rugger-jersey spine appearance consistent with renal osteodystrophy related to chronic kidney disease (Figure 4).

Histopathological examination confirmed ccRCC. No definite distant metastatic disease was identified on the available imaging studies. Considering the locally advanced unresectable disease with extensive venous involvement, palliative systemic therapy was initiated.

## Discussion

RCC is unique among solid malignancies because of its marked propensity for venous invasion. Tumor thrombus develops in approximately 4-10% of patients and most commonly involves the renal vein, with subsequent extension into the IVC in advanced disease. The presence and cranial extent of venous tumor thrombus are major determinants of tumor, node, and metastasis (TNM) staging, operative complexity, and oncological outcomes. Consequently, accurate preoperative assessment is mandatory for appropriate treatment planning [1,2,4].

Collateral venous extension represents a distinctly uncommon pattern of tumor dissemination. The left gonadal vein is particularly susceptible because it drains directly into the left renal vein, providing a potential route for retrograde intraluminal tumor propagation when venous outflow is compromised. Only isolated reports have documented gonadal vein tumor thrombus secondary to RCC, emphasizing the rarity of this finding [5-7].

The most remarkable feature of the present case was the contiguous extension of tumor thrombus into the left hemiazygos vein, while the IVC remained free of tumor. This unusual pattern is anatomically explained by sequential tumor propagation from the left renal vein into the left gonadal vein, followed by the left ascending lumbar vein, and finally the hemiazygos venous system. Although retrograde tumor extension through the gonadal vein has been documented in a limited number of reports [5-7], extension through the ascending lumbar vein into the hemiazygos vein appears to be exceptionally uncommon. To our knowledge, only very limited data describing this collateral venous pathway have been reported.

Contrast-enhanced MDCT played a pivotal role in the diagnosis and staging of our patient. In addition to accurately characterizing the primary renal tumor, CT demonstrated continuity between the enhancing intravascular soft-tissue component and the renal mass, allowing reliable differentiation between tumor thrombus and bland thrombosis. The principal differential diagnosis is bland venous thrombosis. Unlike tumor thrombus, bland thrombus typically does not enhance after contrast administration, does not expand the involved vein, and lacks direct continuity with the primary renal mass. Recognition of these imaging features is essential because management differs substantially between these two entities [3].

Previous studies have demonstrated the excellent diagnostic performance of MDCT for evaluating venous tumor thrombus and guiding surgical planning. Contemporary clinical series have also shown that the extent of venous tumor thrombus strongly influences surgical strategy and oncological outcomes [8,9].

Another noteworthy aspect of this case is its occurrence in a relatively young woman with end-stage renal disease undergoing maintenance hemodialysis. Patients with end-stage renal disease have a substantially increased risk of developing RCC compared with the general population. Nevertheless, dialysis-associated RCC more frequently arises in the setting of acquired cystic kidney disease and often exhibits non-clear cell histological subtypes. The coexistence of chronic hemodialysis, giant clear cell RCC, and extensive collateral venous tumor extension further enhances the educational value of the present case.

From a practical perspective, this report emphasizes that radiological assessment of locally advanced RCC should not be limited to the renal vein and IVC. Systematic evaluation of collateral venous pathways, including the gonadal, ascending lumbar, azygos, and hemiazygos veins, particularly using multiplanar reconstructions, may reveal unexpected sites of tumor extension with important implications for staging, surgical strategy, and multidisciplinary management [3,4,8-10].

## Conclusions

We report an unusual case of giant ccRCC associated with contiguous tumor thrombus extending into the left renal vein, left gonadal vein, left ascending lumbar vein, and hemiazygos vein without IVC invasion. This case highlights the pivotal role of contrast-enhanced MDCT and multiplanar reconstructions in identifying atypical collateral venous extension beyond the conventional renal vein-IVC pathway. Careful evaluation of collateral venous pathways is essential for accurate staging, comprehensive preoperative assessment, and optimal treatment planning in patients with locally advanced RCC.
